# Thermophysiological BioEnergy Index as a Biomarker of Biological Ageing: A Large-Scale Microwave Radiometry Study

**DOI:** 10.3390/diagnostics16131994

**Published:** 2026-06-26

**Authors:** Igor Goryanin, Larion Popov, Alexander Tarakanov, Sergey G. Vesnin, Christoforos Galazis, Batyr Osmonov, Bob Damms, Alexander Losev, Sanja Mogy, Irina V. Goryanin

**Affiliations:** 1 School of Informatics, University of Edinburgh, Edinburgh EH8 9YL, UK; 2 IQANOVA Ltd., Edinburgh EH10 5LZ, UK; 3 MMWR Ltd., Edinburgh EH10 5LZ, UK; popov.larion@yandex.ru (L.P.); vesnin47@gmail.com (S.G.V.); chrisgalazis@gmail.com (C.G.); bob.damms@gmail.com (B.D.); igoryanin@hotmail.com (I.V.G.); 4 Independent Researcher, Rostov-on-Don 344022, Russia; dr-tarakanov@yandex.ru; 5 Department of Bioengineering, Imperial College, London SW7 2AZ, UK; 6 Medical Center, Kyrgyz State Medical Academy Named After I.K. Akhunbaev, Bishkek 720020, Kyrgyzstan; 7 Independent Researcher, Volgograd 400062, Russia; allosev59@gmail.com; 8 Mogy Clinics, 1236 Trzin, Slovenia; sanja@mo-gy.si

**Keywords:** ageing, BioEnergy Index (BEI), microwave radiometry, thermophysiology, mitochondrial function, metabolic decline, biological age, microvascular endothelial dysfunction, autonomic thermoregulation, lobular involution

## Abstract

**Background/Objectives:** Biological ageing is accompanied by progressive alterations in mitochondrial metabolism, microvascular function, and thermoregulation. These processes collectively influence tissue heat production and dissipation, reflecting integrated metabolic, vascular, and thermoregulatory activity measurable at the physiological level. Passive microwave radiometry (MWR) provides a non-invasive, radiation-free method for detecting deep-tissue bioenergy emissions, complementing surface infrared thermography. To evaluate a thermophysiological Bioenergetic Index (BEI), derived from deep-tissue microwave emission, surface temperature, and their spatial and deep–surface relationships, as a candidate age-referenced thermophysiological marker associated with chronological ageing. **Methods:** Breast thermophysiology measurements from 36,391 women aged 20–80 years were analysed using data collected during routine clinical assessments. Supervised machine-learning models were trained exclusively on thermal features, with chronological age used only as the prediction target. Model performance was assessed using mean absolute error (MAE), root mean square error (RMSE), and coefficient of determination (R^2^). In addition, data were aggregated into 5-year age bins to evaluate population-level ageing trajectories. **Results:** At the individual level, models predicted chronological age with MAE ≈ 3.5 years, RMSE ≈ 5.4 years, and R^2^ ≈ 0.76. Aggregation into 5-year age bins revealed a robust nonlinear ageing trajectory characterised by midlife decline and late-life stabilisation. The increased correspondence at the grouped level reflects reconstruction of the population-level ageing trajectory rather than improved individual-level prediction accuracy, as averaging reduces inter-individual variability. **Conclusions:** These findings demonstrate a strong ageing-related signal in female breast thermophysiology and support thermophysiology as a candidate age-referenced physiological marker, pending longitudinal and outcome-based validation. The present analysis is cross-sectional and requires longitudinal validation before diagnostic or prognostic interpretation.

## 1. Introduction

**Figure 1:**
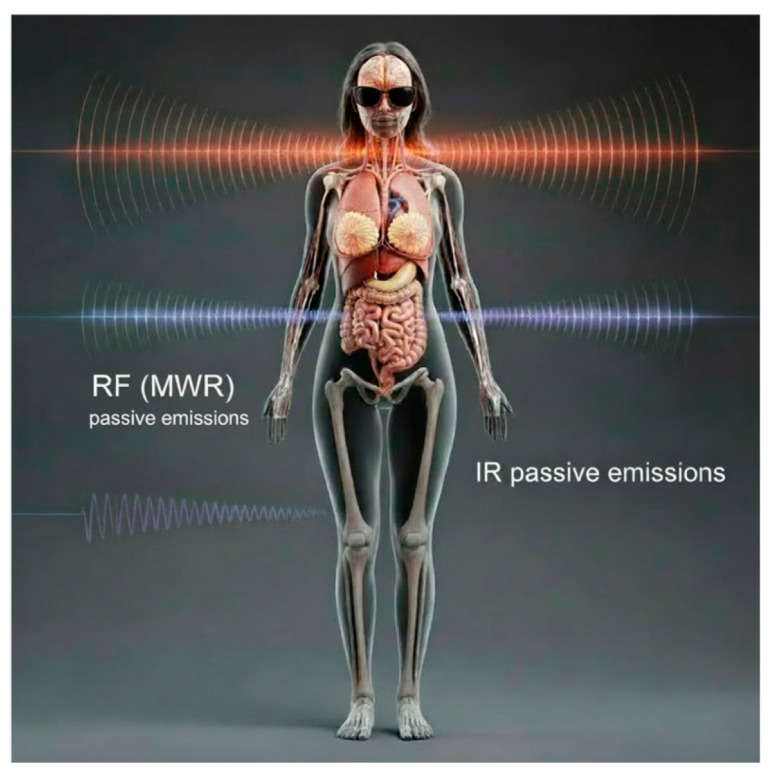
Thermophysiological framework for bioenergetic ageing. Deep-tissue radiofrequency (RF) radiometry captures bioenergy emission associated with metabolic activity and vascular heat transport, whereas infrared (IR) radiometry reflects surface energy dissipation. Ageing is associated with a progressive reduction in deep-tissue bioenergy emission and altered deep–surface energetic gradients, providing a physiologically interpretable basis for biological age estimation, but with an important caveat: the decline is nonlinear. Physiological studies indicate that thermoregulatory efficiency decreases most rapidly in midlife, yet tends to stabilise or plateau after approximately 65–70 years as compensatory mechanisms fail and heat production reaches a lower steady state [7].

Chronological ageing is accompanied by systemic physiological decline affecting metabolism, vascular dynamics, thermoregulation, and inflammatory balance [1]. Existing biological ageing clocks—including DNA methylation clocks, proteomic signatures, metabolomic clocks, and wearable-based physiological models—capture different aspects of ageing, yet they rely either on biochemical assays or long-term sensor data and often remain costly or invasive [2]. In contrast, microwave radiometry (MWR) [3] provides a uniquely non-invasive, radiation-free method to measure deep-tissue emissions, offering indirect physiological insight into tissue thermal state, perfusion dynamics, and heat transport mechanisms and microvascular function—processes tightly linked to ageing biology (Figure 1) [4].

MWR measures natural electromagnetic emission primarily determined by tissue water content, ionic conductivity, and temperature distribution. While mitochondrial metabolism contributes to heat generation, MWR does not directly quantify mitochondrial oxidative phosphorylation, and mechanistic interpretation remains inferential.

We demonstrated that microwave emission can be used for early breast cancer diagnosis [5] and has been explored in prior biomedical applications of microwave radiometry as a non-invasive physiological measurement modality [6].

Ageing is known to reduce basal metabolic heat, decrease brown adipose thermogenesis, impair microvascular tone, and alter autonomic regulation of heat dissipation. Prior work has shown that both core and surface temperatures gradually decline [8,9,10,11,12].

Importantly, thermophysiological signatures are expected to be tissue-, sex- and anatomy-specific. Breast tissue in women represents a unique thermophysiological environment, influenced by hormonal status, microvascular organisation, and adipose composition. Accordingly, the present study is explicitly framed as a proof-of-concept analysis of ageing-related thermophysiological patterns in female breast tissue and does not assume direct generalisability to men or to other anatomical regions.

In this context, we adopt an operational definition of biological age similar to that used in epigenetic and proteomic clocks, in which physiological features are used to predict chronological age, and deviations between predicted and actual age are interpreted as reflecting differences in biological state. While this framework enables discovery of ageing-related physiological structure, it does not, by itself, establish prognostic or causal relevance, which requires longitudinal and outcome-based validation.

Because thermophysiology reflects the integrated function of the metabolic, vascular, and autonomic systems, it provides a promising basis for estimating biological age. However, despite emerging interest, MWR has never been evaluated as an ageing biomarker in a sufficiently large human population nor systematically tested using modern machine-learning approaches. Moreover, the extent to which deep–surface temperature gradients, thermal variance, anatomical scaling, and nonlinear relationships contribute to the ageing signal remains unknown.

Here, we investigate whether deep-tissue and surface thermophysiological features measured by MWR can capture reproducible ageing patterns in a large retrospective cohort of women undergoing routine breast thermophysiology assessments. Our objective is to evaluate the feasibility of a thermophysiological Bioenergetic Index (BEI) as a scalable, non-invasive ageing metric, with particular relevance for translational research and early-phase drug development targeting metabolic, vascular, and inflammatory ageing processes.

## 2. Materials and Methods

**Figure 2:**
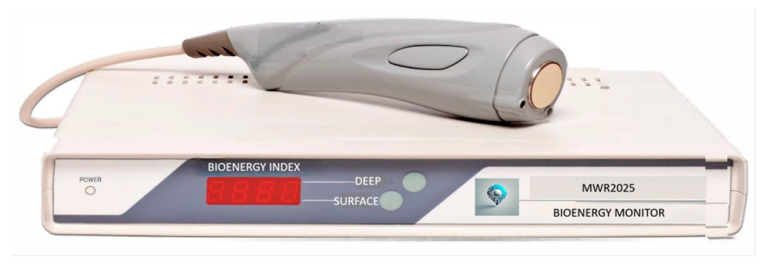
MWR2025. Bioenergy monitor.

This study analysed a large-scale retrospective dataset comprising 44,789 female subjects aged 20–80 years, collected during routine breast thermophysiology assessments in private clinics in Europe using a commercial bioenergy monitor (the new version MWR2025, MMWR LTD, Edinburgh, UK, www.mmwr.co.uk) (Figure 2). Measurements obtained from earlier MWR system versions (hardware is the same) were performed using equivalent acquisition protocols.

All participants underwent non-invasive deep-tissue thermal measurements, surface skin temperature mapping, and breast anatomical assessment. After quality control and exclusion criteria, 36,391 records remained for analysis. Data collected during routine breast thermophysiology assessments; breast pathology status was not systematically recorded, and subclinical disease states may influence thermal measurements.

The inclusion criteria were restricted to female participants to ensure the anatomical and physiological homogeneity of the measured tissue. Incomplete records or those lacking essential thermal or anatomical variables were excluded from the analysis. All measurements were obtained under standardised clinical conditions, including controlled room temperature and uniform sensor placement protocols, to minimise environmental artifacts.

All data were irreversibly anonymised at the point of collection prior to analysis, and no identifiable personal data were accessed by the authors. The study therefore represents retrospective secondary analysis of anonymised clinical measurements collected during routine assessments rather than prospective human-subject research. Data handling complied with applicable European data protection regulations (GDPR). Measurements obtained from earlier MWR system versions underwent device-specific calibration procedures prior to inclusion in the combined dataset to ensure consistency of temperature estimates across hardware generations.

**Figure 3:**
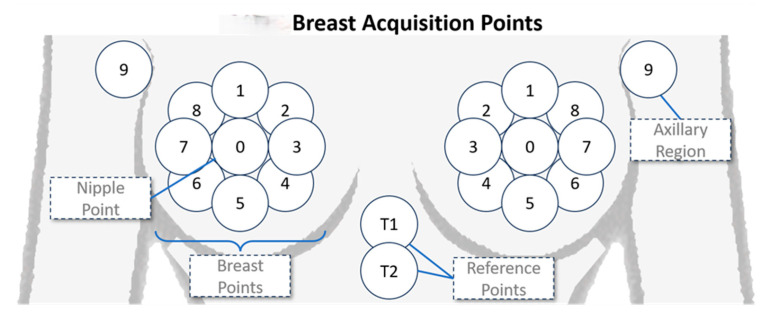
Breast bioenergy acquisition points.

Passive Microwave Radiometry (MWR) was employed to assess internal tissue thermodynamics. Unlike infrared thermography (IRT), which is limited to detecting cutaneous thermal emission, MWR is a non-ionising technique capable of detecting natural electromagnetic emissions at microwave frequencies. The MWR2025 system operates in the microwave frequency range of approximately 3.4–4.2 GHz, enabling estimation of internal tissue temperature. For each participant, 22 points on the breast and axillary areas were recorded (Figure 3). The system incorporates internal electromagnetic shielding and band-pass filtering to minimise environmental electromagnetic interference within the operating frequency range. Measurements were conducted in standard clinical examination rooms free from high-power microwave sources. Each of the 22 acquisition points required approximately 5–10 s of signal stabilisation, resulting in a total acquisition time of approximately 5 min per subject under standard workflow conditions.

Skin emissivity for surface thermistor measurements was assumed to be consistent with standard dermatological thermography practice (emissivity ≈ 0.98). Deep-tissue microwave radiometry relies on natural electromagnetic emission and does not require optical emissivity correction. Depth estimation reflects effective sensitivity across a weighted tissue volume (approximately 3–7 cm) and does not represent discrete layer separation within a single measurement.

Breast diameter (mm) was recorded for each participant as an anatomical modifier of thermal dynamics. Variations in tissue volume can alter heat diffusion, insulation properties, and deep–surface temperature gradients; therefore, diameter was included to account for anatomical scaling effects in the predictive modeling. However, breast diameter is not a validated surrogate for breast tissue composition or lobular involution status. Body mass index (BMI) and detailed body composition parameters were not available and may influence thermal insulation and surface temperature variability; this represents an additional source of unexplained variance. Because menopausal status, hormone therapy use, and detailed adipose phenotyping were not recorded, endocrine and compositional contributions to thermophysiological ageing patterns cannot be disentangled from chronological ageing effects in the present retrospective dataset.

To capture the nonlinear relationship between thermophysiology and chronological age, we developed an age-stratified energetic calibration framework. The Bioenergetic Index (BEI) is a composite score derived from multiple thermophysiological features extracted from microwave radiometry measurements, including mean deep-tissue temperature, spatial variability of deep-tissue temperature, bilateral symmetry, and coupling between deep and surface temperatures. Each feature was normalised across the cohort and combined using supervised machine-learning-derived weights to produce a dimensionless index scaled from 0 to 100. The BEI was optimised to capture age-associated thermophysiological organisation rather than local temperature values.

BEI should therefore be interpreted as a learned, multivariate representation of thermophysiological state. This design allows BEI to capture nonlinear interactions between metabolic heat generation, spatial heterogeneity, vascular symmetry and deep–surface coupling, which would be lost in univariate or pointwise temperature metrics. Because the BEI is calibrated against age-stratified energetic references, it does not assume linear dependence on chronological age. The BEI formula is:

BEI = 100 × (A_ma_^x^ − A_p_^hys^)/(A_ma_^x^ − A_opt_)

where A_p_^hys^ is the model-derived thermophysiological age estimate, A_opt_ is the optimal energetic reference (near 25 years), and A_ma_^x^ is the late-life energetic plateau (mean A_p_^hys^ for ages ≥ 65).

All data processing and modeling were performed in a secure computational environment using Python 3.10. Ensemble tree-based methods (Random Forest and gradient boosting) implemented in Scikit-learn 1.9 were used to capture nonlinear interactions. Visualisations were generated using scatter plots with locally weighted regression (LOESS) smoothing and age-bin aggregation plots using NumPy 1.25, pandas 3.0, and Matplotlib 3.11.0.

## 3. Results

The initial dataset contained 44,789 records. After restricting ages to the physiologically stable range of 20–80 years and removing multivariate outliers beyond ±3 SD across deep and surface temperatures, breast diameter, and control points, 36,391 high-quality samples remained. Model performance was evaluated across breast diameter categories, and prediction error remained comparable across size strata, indicating robustness to anatomical scaling within the studied cohort.

Age binning was performed exclusively for post hoc visualisation and reconstruction of the population-level ageing trajectory. All supervised models were trained using continuous chronological age as the target variable; bin-level averages were never used as training labels.

### 3.1. BEI Trends with Age

**Figure 4:**
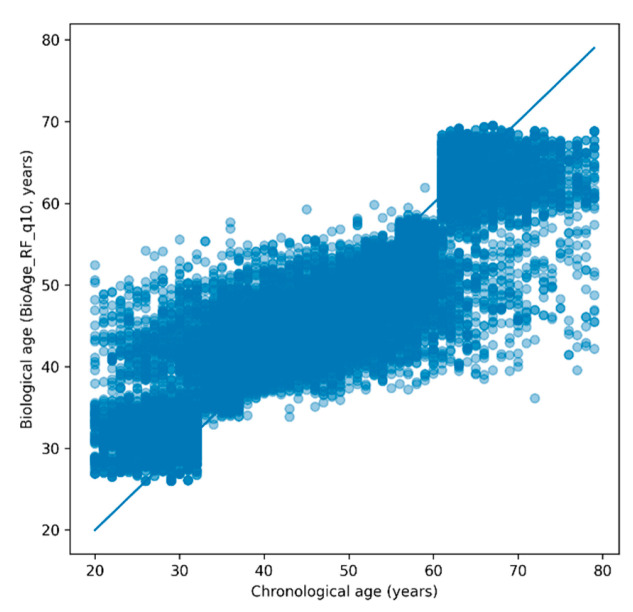
Bioenergetic Index (BEI) across age. Mean ± SEM BEI values by age-bin confirm normalisation behaviour and post-65 stabilisation.

Analysis of both raw and age-binned temperatures revealed a distinctly nonlinear relationship between thermophysiology and age. In early adulthood (before ~25–30 years), BEI values tended to increase, consistent with maturation and optimisation of mitochondrial, vascular, and autonomic function. Between ~30 and 55 years, deep-tissue BEI declined progressively (Figure 4), in line with expected age-related reductions in mitochondrial heat production, microvascular perfusion, and autonomic regulation of thermal balance. Surface (skin) temperatures showed a broadly similar downward trend, but with greater person-to-person variability, reflecting their higher sensitivity to environmental and behavioural factors.

Beyond approximately 65 years of age, the BEI trajectory flattened, with only minimal additional decline. This late-life plateau was evident in both individual-level data and 5-year age-bin averages. The presence of such a plateau suggests a shift to a thermophysiological steady state, in which further reductions in metabolic output are constrained and variability is suppressed—likely reflecting a combination of reduced metabolic flexibility, diminished vascular reactivity, and narrowed autonomic range in very late life [13]. Feature importance analysis (SHAP-based ranking) demonstrated that deep–surface energetic gradients and mean deep-tissue temperature were the dominant contributors to chronological age prediction, whereas surface temperature alone contributed less strongly due to higher environmental variability. Correlation analysis indicated that deep-tissue RF-derived temperatures and surface IR measurements were moderately correlated but not collinear, supporting the interpretation that RF-derived deep-tissue signals capture metabolic heat generation partially independent of surface dissipation dynamics.

### 3.2. Biological Age Prediction

Using chronological age as the supervisory signal (target) but never as an input, we trained supervised regression models (Random Forest and gradient-boosted trees) to predict age from BEI and its constituent features. The model-derived value ${\hat{A}}_{i}$ is defined as the thermophysiological age estimate (BioAge), an age-referenced construct derived from thermal features. Because the mapping is learned purely from thermophysiology, BioAge reflects the age that best matches a subject’s thermophysiological profile within the learned age-referenced model space, rather than serving as a validated prognostic biological age measure.

A critical aspect of our methodology was the exclusion of chronological age from the feature set. Chronological age was used exclusively as the target label (ground truth) for training. The input features consisted solely of the thermophysiological variables, BEI and anatomical diameter. The dataset was split into training (80%) and testing (20%) subsets using stratified sampling to maintain age distribution balance.

**Figure 5:**
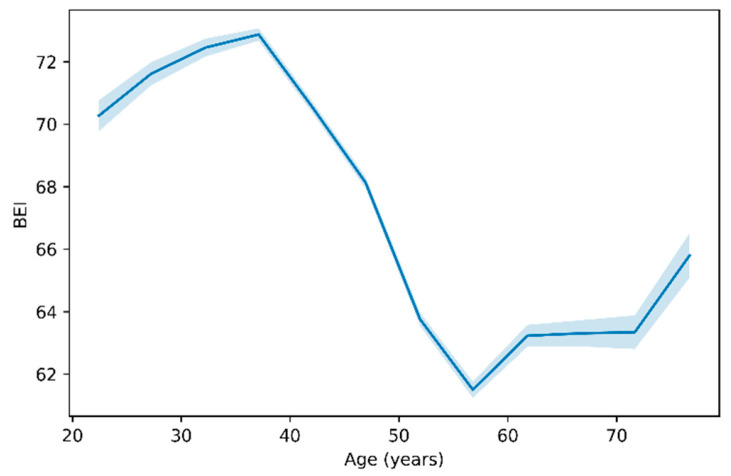
The predicted individual biological age against chronological age. Model performance metrics: RMSE ≈ 5.37 y, MAE ≈ 3.53 y, R^2^ ≈ 0.757.

The Random Forest regression model predictions are off by about 3.5 years (MAE). Squared-error-weighted deviation is about 5.4 years (RMSE); the model explains about 76% of the variance in chronological age across 20–80 years using deep MWR + skin temperature features (Figure 5).

Aggregation into 5-year age bins gives R^2^ ≈ 0.984, RMSE ≈ 1.38 years. This apparent improvement reflects statistical noise reduction through averaging rather than enhanced individual-level predictive accuracy. Individual-level metrics (MAE ≈ 3.5 years; R^2^ ≈ 0.76) represent the primary measure of model performance.

Given the nonlinear relationship between thermophysiological energy and age, we evaluated Random Forest models trained within age strata (<30, 30–65, ≥65 years). Stratified models substantially reduced prediction error (RMSE 3.8–6.2 years), indicating that age-specific physiological regimes contribute distinct energetic signatures. The very low within-stratum R^2^ values (0.003–0.081) reflect a known statistical property of regression within narrow target ranges; RMSE provides a more appropriate performance metric within strata (Table 1). 

### 3.3. MWR-NetAge

In addition to the Random Forest model, we trained an MWR-based neural network (MWR-NetAge) both with and without 5-year age binning. The architecture utilises four MWR block layers with a hidden dimension of 256 using ReLU activation functions, adapted for regression. To improve robustness, five MWR-NetAge models were trained, each on a random 50% subsample of the training data; final predictions were averaged across this ensemble.

Without age binning, MWR-NetAge achieves RMSE ≈ 4.82 years, MAE ≈ 3.38 years, and R^2^ ≈ 0.801. When trained with 5-year age bins, performance improves to RMSE ≈ 1.21 years, MAE ≈ 1.02 years, and R^2^ ≈ 0.987. As with Random Forest models, bin-level performance reflects post hoc averaging rather than improved individual prediction.

## 4. Discussion

### 4.1. The Biological Meaning of BEI Ageing

Deep-tissue bioenergy emission represents an integrative physiological quantity. It reflects the balance between cellular heat production and vascular heat dissipation, regulated by mitochondrial oxidative phosphorylation, microvascular vasomotion and perfusion, autonomic nervous system tone, systemic inflammatory status, and endocrine–metabolic state.

The deep–surface energetic gradient captured by BEI integrates two physiologically distinct axes of ageing. The first is metabolic: mitochondrial oxidative phosphorylation in glandular and stromal cells generates heat constituting the principal source of deep-tissue bioenergy emission measurable by MWR. Age-related mitochondrial dysfunction—including reduced complex I/III activity, increased uncoupling, and loss of mitochondrial mass—progressively reduces this heat generation, lowering deep-tissue MWR signal. The second axis is vascular–autonomic: transport and dissipation of metabolically generated heat through the breast depends critically on microvascular perfusion and its sympathetic nervous system regulation. Endothelial dysfunction—characterised by reduced nitric oxide bioavailability, impaired flow-mediated dilation, and increased oxidative stress—reduces vasodilatory reserve and impairs heat removal from deep tissue, altering the deep–surface gradient. Simultaneously, age-related decline in sympathetic vasomotor tone, baroreceptor sensitivity, and autonomic heart rate variability reduces the dynamic range of cutaneous vasoconstriction and vasodilation, narrowing the physiological bandwidth of surface heat dissipation. Together, these two axes—declining metabolic heat production and impaired autonomic–endothelial heat transport—produce the composite thermophysiological signature captured by BEI. Critically, neither axis alone is sufficient: the deep–surface gradient emerged as the dominant predictor in SHAP-based feature importance analysis, consistent with the interpretation that BEI captures the coupling between metabolic generation and vascular/autonomic dissipation. This dual-axis framing positions BEI as a non-invasive integrative readout of two of the most clinically important ageing axes: mitochondrial metabolic decline and vascular–autonomic dysfunction.

As individuals age, all of these regulatory axes undergo progressive impairment. Mitochondrial efficiency declines, reducing basal metabolic heat output; microvascular stiffening and endothelial dysfunction impair the distribution and removal of heat; and age-related changes in autonomic control diminish the ability to adapt thermoregulation to internal and external demands. Together, these changes drive a gradual but measurable fall in both deep and surface bioenergy emissions, detectable with high sensitivity by MWR.

In our dataset, these processes manifested as a distinctly nonlinear thermophysiological ageing pattern. We observed (i) a rapid decline in BEI between approximately 30 and 55 years, consistent with metabolic slowdown, perimenopausal transition, and microvascular ageing, and (ii) a marked stabilisation after about 65–70 years, suggesting a thermophysiological plateau in which further reductions in metabolic heat output are constrained by a minimal baseline state.

Feature importance analyses showed that the strongest predictors were deep-tissue temperatures and deep–surface energetic gradients, rather than surface readings alone. Deep temperatures primarily index true metabolic heat generation and are comparatively insulated from ambient influences, while deep–surface gradients quantify how efficiently heat is transported and dissipated through skin and subcutaneous tissues, processes governed by vascular integrity and autonomic regulation.

### 4.2. Comparison to Existing Biological Ageing Methods

First-generation epigenetic clocks such as the Horvath multi-tissue estimator and the Hannum blood-based predictor typically achieve R^2^ in the ~0.70–0.90 range for chronological age prediction, with age errors of ~3–7 years [9,14]. Second-generation models such as DNAm, PhenoAge, and GrimAge improve associations with morbidity and mortality, but still rely on relatively expensive, batched laboratory assays [15,16,17]. Proteomic and metabolomic clocks report broadly similar performance (R^2^ ~0.65–0.90, RMSE ~4–8 years) while providing rich mechanistic information [16,17,18]. Wearable-derived clocks report R^2^ ~0.4–0.6, RMSE often 5–10 years [19].

Against this backdrop, the thermophysiological BEI achieves individual-level performance (R^2^ ≈ 0.8; RMSE ≈ 4.8 years across 20–80 years) comparable to first-generation biological age clocks in terms of chronological age prediction accuracy. Unlike molecular clocks, BEI is obtained from a single, non-invasive microwave/infrared measurement requiring no blood draw, no laboratory processing, and no longitudinal tracking. Compared with DNA methylation sequencing, passive microwave radiometry requires only device amortisation and routine calibration, without consumables or laboratory infrastructure.

Performance comparison refers only to chronological age prediction accuracy. Validation scope differs substantially: the BEI is not outcome-validated. Modalities are complementary, not competitive (Table 2).

**Table 2:** Comparison of different ageing clock methods.

Clock Type	Typical R^2^	RMSE (Years)	Key Limitations
DNA methylation clocks (Horvath, Hannum, PhenoAge)	0.70–0.92	3–7	Costly lab tests; batch effects; delayed readout
Proteomic/metabolomic clocks	0.65–0.90	4–8	Highly invasive, expensive, assay-dependent
Wearable-derived clocks (HRV, sleep, activity)	0.40–0.65	5–10	High noise; long-term tracking required
Thermophysiological BEI (this work)	≈0.8	≈4.8	Local tissue signal; requires controlled setup; breast-specific; cross-sectional only

### 4.3. Clinical and Diagnostic Implications

**Figure 6:**
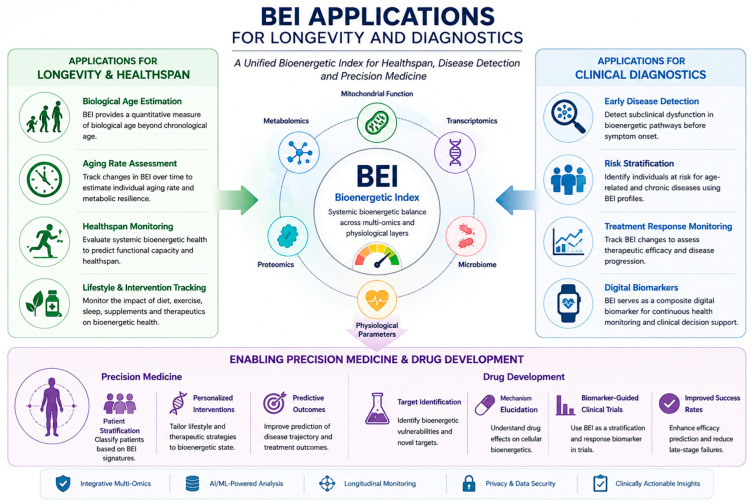
BEI applications for longevity and diagnostics.

The ability to estimate biological age from thermophysiology with clinically meaningful accuracy has direct implications for preventive health and early risk stratification (Figure 6). A positive thermophysiological Age Delta (BioAge ≫ chronological age) may hypothetically identify individuals with altered thermophysiological profiles, a possibility requiring prospective validation. Microvascular and autonomic dysfunction are among the earliest manifestations of cardiometabolic disease, and both are tightly coupled to skin and deep-tissue thermal responses.

Thermophysiological biological age is also attractive as a dynamic readout for lifestyle and pharmacological interventions. Because deep-tissue heat production and microvascular control are directly modulated by mitochondrial activity, autonomic tone, and inflammatory state, thermal ageing is likely to be sensitive to exercise-induced mitochondrial activation, dietary optimisation, anti-inflammatory therapies, and hormone-modulating treatments. A non-invasive BEI measurement may in future be evaluated for repeated measurement applications, pending longitudinal validation.

### 4.4. Nonlinearity and Physiological Interpretation

The recovery of a nonlinear ageing trajectory—with a steep midlife decline followed by a late-life plateau—without imposing this shape a priori provides strong evidence that the thermophysiological model is consistent with known biological ageing patterns. The marked midlife fall in deep and surface temperatures is consistent with converging evidence for age-related impairments in mitochondrial quantity and quality [16,17]. In women, the menopausal transition superimposes a loss of estrogen-mediated vasodilation and endothelial protection on the background of vascular ageing [18,19]. During the perimenopausal transition, reduced estrogen-mediated vasodilation may attenuate heat transport efficiency, potentially increasing deep–surface gradient magnitude despite declining absolute temperatures.

The late-life thermal plateau (≥65–70+ years) can be interpreted as a transition to a constrained but relatively stable thermodynamic regime. Alternative explanations include survivorship bias (enrichment for physiologically resilient individuals), age-related breast tissue involution altering thermal properties, and potential sensitivity limits of thermophysiological measurements at lower temperature ranges. Because the dataset is cross-sectional, causal interpretation of late-life stabilisation is not possible. The late-life plateau should not be interpreted as direct equivalence to basal metabolic rate (BMR), which reflects whole-body calorimetric energy expenditure; rather, it represents a stabilised local tissue thermodynamic regime measurable via MWR. Thermal spatial variance tended to decrease modestly with advancing age, consistent with reduced autonomic flexibility and diminished physiological complexity in late life.

The nonlinear thermal trajectory aligns with modern theories of ageing emphasising (i) metabolic vitality decline driven by mitochondrial dysfunction and loss of thermogenic tissues [16,17,20,21,22]; (ii) vascular-first ageing, in which micro- and macrovascular deterioration precede overt organ failure [18,19]; and (iii) loss-of-variability or complexity, in which homeostatic control becomes increasingly constrained with advancing age [23,24].

### 4.5. Strengths and Limitations

This work introduces a novel, fully non-invasive thermophysiological framework for quantifying age-related metabolic and microvascular changes using passive microwave radiometry. A major strength is the combination of deep-tissue temperature measurements with a very large, real-world clinical cohort (*N* > 36,000, ages 20–80), which enables robust characterisation of both individual variability and the nonlinear population ageing trajectory. The modelling strategy is explicitly physiology-driven, and the models are trained without chronological age as an input, minimising trivial self-prediction.

There are, however, several important limitations. The dataset is retrospective and derived from routine breast imaging in women, so clinical conditions were not fully standardised. The cohort is female-only and breast-specific, meaning that the inferred thermophysiological patterns cannot be assumed to generalise to men or to other anatomical regions. Because the cohort derives from women undergoing breast assessment, health-seeking bias cannot be excluded. The analysis is cross-sectional, precluding direct inference on intra-individual trajectories or causality. Systematic data on metabolic disease, cardiovascular outcomes, and inflammatory biomarkers were not available, restricting evaluation of Age Delta as a prognostic marker. Repeated measurements within the same individual were not available; therefore, intra-individual stability of BEI across time could not be assessed.

Age-related lobular involution represents an important anatomical confounder of breast thermophysiological signals. As glandular tissue is progressively replaced by adipose and fibrous stroma across the adult lifespan, the thermal conductivity, heat generation capacity, and deep–surface gradient structure of breast tissue change independently of systemic metabolic ageing. The present dataset does not include imaging-based breast composition measures (mammographic density, MRI glandular volume, or ultrasound tissue characterisation), making it impossible to statistically separate involution-driven thermophysiological changes from those attributable to metabolic, vascular, and autonomic ageing processes. Future studies should incorporate validated breast composition measures as covariates in the BEI prediction model. Specifically, a regression framework including mammographic density category or MRI-derived fibroglandular tissue fraction alongside thermophysiological features would allow partial separation of involution and ageing contributions to the observed BEI trajectory. Until such analyses are available, the BEI trajectory should be interpreted as reflecting the integrated thermophysiological state of the breast—encompassing both anatomical and physiological ageing—rather than a purely systemic metabolic ageing signal.

Importantly, because breast pathology status, menopausal state, and systemic inflammatory markers were not systematically recorded, the observed thermophysiological age trajectory may partially reflect endocrine transitions, tissue involution, or subclinical disease processes rather than organismal ageing alone. Prospective studies with richer phenotyping will be required to disentangle these contributions. The BEI-derived biological age should therefore be interpreted as an age-referenced physiological construct rather than an outcome-validated biomarker of healthspan or longevity.

Key Strengths

- Very large clinical dataset (*N* > 36,000) spanning ages 20–80;
- High-quality, standardised deep-tissue and surface thermal measurements;
- Fully non-invasive, low-risk and operationally scalable modality;
- Physiology-driven feature engineering (deep, surface, gradients, heterogeneity, BEI);
- Predictive models trained without chronological age as an input feature;
- Robust reconstruction of both individual BioAge and the nonlinear population-level ageing trajectory;
- Dual-axis mechanistic framework: mitochondrial metabolic heat generation, and vascular–autonomic heat transport/dissipation.

Key Limitations

- Female-only, breast-imaging cohort; generalisation to mixed-sex populations and other anatomical regions remains to be demonstrated;
- Retrospective, cross-sectional design rather than longitudinal follow-up;
- Limited clinical annotation; lack of systematic metabolic, cardiovascular and inflammatory phenotypes to correlate with Age Delta;
- Breast-specific anatomy and thermal field geometry; lobular involution as uncontrolled anatomical confound;
- No imaging-based breast composition data to statistically separate involution-driven from ageing-driven thermophysiological changes;
- Menopausal status, hormone therapy use, and adipose phenotyping not available.

### 4.6. Future Work

Several lines of work follow naturally from these findings. First, the present analysis is restricted to breast measurements in women; extending thermophysiological assessment to other anatomical regions (e.g., abdomen, back, extremities, brain/skull) will be important. Second, longitudinal studies are needed to quantify the stability and reversibility of Age Delta—specifically, whether individuals with accelerated thermophysiological ageing can be shifted towards a younger profile by lifestyle, pharmacological, or device-based interventions. Third, integrating thermophysiology with molecular and digital biomarkers represents a promising direction. Fourth, incorporating breast composition imaging measures (mammographic density, MRI fibroglandular tissue fraction) as covariates in future BEI models is a priority to disentangle involution-driven from metabolic ageing-driven thermophysiological changes. Fifth, targeted studies in men, high-performance athletes and clinically defined subcohorts (metabolic syndrome, autoimmune disease, oncology, menopause clinics) will be essential.

A prospective validation study would require longitudinal follow-up of a thermophysiologically phenotyped cohort with linkage to mortality, cardiovascular events, metabolic outcomes, and frailty indices over 5–10 years. Cox proportional hazards modelling could then assess whether positive thermophysiological Age Delta independently predicts adverse outcomes after adjustment for chronological age and conventional risk factors.

## 5. Conclusions

This study demonstrates that deep-tissue thermophysiology, summarised by a Bioenergetic Index (BEI), supports thermophysiology as a promising non-invasive physiological marker of age-related change. Using data from 36,391 women aged 20–80 years, machine-learning models were trained on thermophysiological features alone—deep-tissue temperature, skin temperature, deep–surface gradients, and thermal variance—explicitly excluding chronological age as an input. At the individual level, the resulting biological age estimate showed a mean absolute error of ~3.5 years and an RMSE of ~5.4 years (R^2^ ≈ 0.76). At the population level, the average thermophysiological ageing trajectory across 5-year age bins closely reproduced the chronological ageing curve (R^2^ = 0.984, RMSE = 1.38 years). This level of performance is comparable in chronological age prediction accuracy to first-generation clocks, while differing substantially in modality, tissue specificity and validation scope.

The recovered ageing curve is inherently nonlinear, with a pronounced midlife decline in deep-tissue heat production and vascular thermoregulation between approximately 30 and 55 years, followed by a stabilisation plateau after about 65–70 years. This trajectory is consistent with known age-related changes in mitochondrial function, microvascular stiffness, endocrine transitions, and diminished autonomic flexibility. The dominant predictors—deep-tissue bioenergy emissions and deep–surface energetic gradients—reflect the dual-axis nature of BEI, simultaneously capturing mitochondrial metabolic decline and vascular–autonomic dysfunction, two central and clinically important hallmarks of physiological ageing.

The BEI-derived biological age is an age-referenced thermophysiological construct calibrated against chronological age. It should be understood as a proof-of-concept physiological biomarker rather than an outcome-validated measure of healthspan or longevity. Prospective longitudinal validation against independent outcomes (mortality, cardiovascular events, frailty) is required before clinical application. Critically, because breast-specific lobular involution and menopausal endocrine transitions were not controlled for in this retrospective dataset, the observed thermophysiological ageing trajectory reflects the integrated state of the breast—encompassing anatomical and physiological ageing—and cannot be attributed exclusively to systemic metabolic ageing.

As the field moves towards accessible and scalable biomarkers of ageing, bioenergetic thermophysiology offers an attractive, cost-effective modality that aligns well with the practical needs of real-world preventive medicine and early-phase drug development. Future work incorporating breast composition imaging, longitudinal follow-up, multi-site cohorts, and expanded anatomical coverage will be essential to establish the full scope and clinical utility of this approach.

## Figures and Tables

**Table 1:** Performance of Random Forest models trained and evaluated within age strata. Because chronological age variance is intentionally restricted within each stratum, R^2^ becomes unstable; RMSE provides a more informative metric within strata.

Age Group	*N* (Subjects)	RMSE (Years)	R^2^
<30 years	9845	4.92	0.003
30–65 years	24,464	6.18	0.081
≥65 years	2081	3.81	0.009

## Data Availability

All datasets generated and analysed in this study are provided as Supplementary Material. Further technical details and scripts can be provided upon reasonable request.

## Supplementary Materials

The following supporting information can be downloaded at: https://www.mdpi.com/article/10.3390/diagnostics16131994/s1, Table S1: Sensitivity analysis of BEI performance across menopausal status, hormone therapy use, and body-type categories in the Slovenian validation cohort.
