# Supplementary material for: Thermophysiological BioEnergy Index as a Biomarker of Biological Ageing: A Large-Scale Microwave Radiometry Study
Source: Diagnostics (Basel). 2026 Jun 26;16(13):1994. doi: 10.3390/diagnostics16131994 (PMC13359716; doi:10.3390/diagnostics16131994)
Supplement: Supplementary file 1 [file diagnostics-16-01994-s001.zip › diagnostics-4273541-supplementary.pdf]

Supplementary Table S1. Sensitivity analysis of BEI performance across menopausal status, hormone therapy use, and body-type categories in the Slovenian validation cohort.

Subgroup	N	Mean Age (years)	RMSE (years)	MAE (years)	R^2	Principal Observation
Premenopausal	4,238	42.1 ± 7.3	4.9	3.4	0.77	Nonlinear trajectory preserved
Postmenopausal	4,762	57.4 ± 6.9	6.1	4.1	0.75	Similar trajectory shape; higher RMSE attributable to older age distribution
Hormone therapy users	982	56.1 ± 6.4	5.8	3.9	0.76	No material alteration of trajectory shape observed
No hormone therapy	4,762	53.8 ± 9.1	5.5	3.8	0.76	Comparable performance
Very thin	624	44.6 ± 10.5	5.2	3.5	0.76	Slight upward shift in thermal peak age
Thin	1,984	47.8 ± 9.8	5.3	3.6	0.76	Preserved trajectory
Normal weight	3,216	50.9 ± 10.1	5.4	3.7	0.77	Reference pattern
Overweight	2,102	54.7 ± 8.8	5.6	3.8	0.76	Earlier trajectory peak
Obese	1,074	56.2 ± 8.1	5.8	4.0	0.75	Preserved trajectory shape with age-shifted maximum

Table Legend

Supplementary Table S1. Sensitivity analysis of the BioEnergy Index (BEI) ageing model across menopausal status, hormone therapy use, and body-type categories in an independent Slovenian validation cohort. Across all subgroups, the characteristic nonlinear BEI ageing trajectory (midlife decline followed by late-life stabilisation) remained qualitatively preserved. Differences in RMSE between menopausal groups were largely explained by differences in age distribution rather than menopausal status itself. Increasing body mass was associated with a modest shift in the thermophysiological peak toward younger ages but did not materially alter overall trajectory shape. These findings support the robustness of the BEI ageing signal across major physiological subgroups while highlighting the need for prospective studies incorporating formal BMI, hormonal, and body composition measurements.
